# Critical Function of γH2A in S-Phase

**DOI:** 10.1371/journal.pgen.1005517

**Published:** 2015-09-14

**Authors:** Eva Mejia-Ramirez, Oliver Limbo, Petra Langerak, Paul Russell

**Affiliations:** Department of Cell and Molecular Biology, The Scripps Research Institute, La Jolla, California, United States of America; Duke University, UNITED STATES

## Abstract

Phosphorylation of histone H2AX by ATM and ATR establishes a chromatin recruitment platform for DNA damage response proteins. Phospho-H2AX (γH2AX) has been most intensively studied in the context of DNA double-strand breaks caused by exogenous clastogens, but recent studies suggest that DNA replication stress also triggers formation of γH2A (ortholog of γH2AX) in *Schizosaccharomyces pombe*. Here, a focused genetic screen in fission yeast reveals that γH2A is critical when there are defects in Replication Factor C (RFC), which loads proliferating cell nuclear antigen (PCNA) clamp onto duplex DNA. Surprisingly Chk1, Cds1/Chk2 and the Rad9-Hus1-Rad1 checkpoint clamp, which are crucial for surviving many genotoxins, are fully dispensable in RFC-defective cells. Immunoblot analysis confirms that Rad9-Hus1-Rad1 is not required for formation of γH2A by Rad3/ATR in S-phase. Defects in DNA polymerase epsilon, which binds PCNA in the replisome, also create an acute need for γH2A. These requirements for γH2A were traced to its role in docking with Brc1, which is a 6-BRCT-domain protein that is structurally related to budding yeast Rtt107 and mammalian PTIP. Brc1, which localizes at stalled replication forks by binding γH2A, prevents aberrant formation of Replication Protein A (RPA) foci in RFC-impaired cells, suggesting that Brc1-coated chromatin stabilizes replisomes when PCNA or DNA polymerase availability limits DNA synthesis.

## Author Summary

ATM (ataxia telangiectasia mutated) and ATR (ATM and Rad3 related) are evolutionary conserved protein kinases that phosphorylate the carboxyl-tail of histone H2AX in chromatin flanking DNA lesions. Phosphorylated histone H2AX (aka γH2AX) tethers important DNA damage response (DDR) proteins to DNA double-strand breaks but its function during DNA replication is unclear. A novel genetic screen reveals that a partial defect in Replication Factor C (RFC) creates a critical requirement for γH2AX in fission yeast. These studies indicate that γH2AX stabilizes replication forks by recruiting Brc1 when RFC is unable to load the DNA clamp known as proliferating cell nuclear antigen (PCNA) onto duplex DNA. Surprisingly, this activity of γH2AX is more critical than ATM/ATR-mediated activation of the checkpoint kinase Chk1 and Chk2.

## Introduction

DNA lesions elicit highly orchestrated DNA damage responses (DDRs) controlled by the master checkpoint kinases ATM and ATR. These responses protect genome integrity and prevent diseases characterized by chromosome instability and cancer [1,2]. ATM and ATR have many substrates but none is more ubiquitous than the SQ motif at the carboxyl tail of histone H2AX or H2A [3]. Key DDR proteins such as mammalian MDC1 have C-terminal regions consisting of tandem BRCA1 C-terminus (BRCT) domains that form a highly sculpted binding pocket for the phosphorylated C-terminus of phospho-H2AX (γH2AX) [4]. These DDR proteins decorate large chromatin domains flanking DNA lesions. However, H2AX phospho-site mutations generally cause modest genotoxin sensitivity compared to eliminating γH2AX-binding proteins, suggesting that docking to γH2AX enhances but is not always essential for DDR protein functions [5–7]. Endogenous sources of DNA damage might create a more acute requirement for γH2AX to protect genome integrity.

Whilst γH2AX has been most intensively studied in the context of DNA double-strand breaks (DSBs) formed by exogenous clastogens, recent studies with fission yeast and budding yeast established that γH2AX (aka γH2A in yeast) increases every DNA synthesis (S)-phase [8,9]. Single-stranded DNA (ssDNA) at stalled or damaged replication forks appears to be the triggering DNA structure. Here, we investigate the function of γH2AX by using a genetic screen to identify DNA replication mutants whose viability critically depends on γH2A in *Schizosaccharomyces pombe*. These studies reveal that a defect in Replication Factor C (RFC), which loads the replicative DNA polymerase processivity factor known as proliferating cell nuclear antigen (PCNA) onto duplex DNA, creates an acute requirement for γH2A. Our studies track this requirement to Brc1, a γH2A-binding protein that functions in the replication stress response [10,11]. From our studies we propose that large-scale adornment of γH2A-marked chromatin with Brc1 prevents replication fork collapse when PCNA loading or DNA polymerase activity limit DNA synthesis.

## Results

### Mutation of Rfc3 creates a critical requirement for γH2A

**Fig 1:**
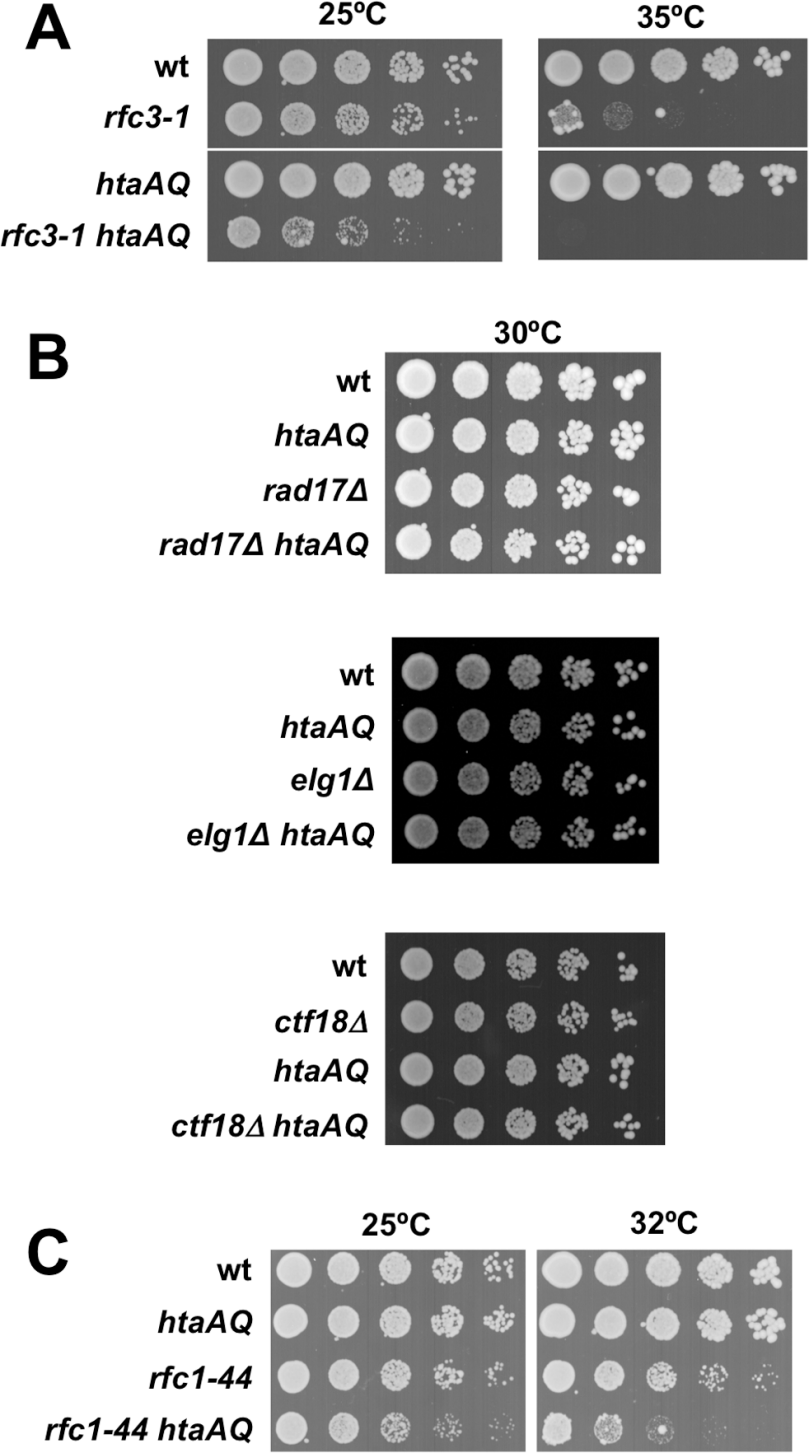
Critical requirement for γH2A when RFC function is impaired. **(A)** The *rfc3-1* and *htaAQ* mutations have a SSL genetic interaction. Tenfold serial dilution of wild type (wt), *rfc3-1*, *htaAQ* (*hta1-S129A hta2-S128A*), and *htaAQ rfc3-1* strains were incubated at permissive (25°C) and restrictive temperatures (35°C). Growth of *htaAQ rfc3-1* cells at 25°C is substantially impaired relative to *rfc3-1* cells. **(B)** Mutations that eliminate alternative RFCs do not have SSL genetic interactions with *htaAQ* mutations. The *rad17∆*, *ctf18∆ and elg1∆* mutations that eliminate large subunits of alternative RFCs were mated into the *htaAQ* background. Growth was assessed at 30°C. **(C)** The *rfc1-44* and *htaAQ* mutations have a SSL genetic interaction.

We have constructed *S*. *pombe* “*htaAQ*” strains in which both histone H2A genes have been mutated to alter the C-terminal SQ phosphorylation site to AQ (*hta1-S129A hta2-S128*), thereby eliminating γH2A [7]. We sought to identify mutations having synthetic sick or lethal (SSL) genetic interactions with *htaAQ*. We used tetrad analysis to introduce *htaAQ* into strains having conditional mutations in genes that are essential for DNA replication. We initially chose mutations of genes encoding subunits of the pre-initiation complex (pre-IC; *sld3-10* and *cdc45-192*), pre-replication complex (pre-RC; *cdc18-K9*), MCM replicative DNA helicase (*mcm2-P1* and *mcm6-568*), Dpb11 replication and checkpoint scaffold protein (*cut5-T401*), replication factor C subunit 3 (*rfc3-1*), and an *Schizosaccharomyces*-specific gene whose product associates with Dna2 flap endonuclease/helicase that is required for Okazaki fragment processing (*cdc24-M28*). For all but one of these mutations the SSL interactions were undetectable or weak when tested in the absence of exogenous DNA damaging agents or replication inhibitors. The most obvious exception was *rfc3-1* [12], which had a clear SSL interaction with *htaAQ* at the permissive temperature of 25°C (Fig 1A). γH2A is therefore critical when Rfc3 function is impaired.

### The requirement for γH2A is specific for defects in RFC

Rfc3 is as an essential subunit of RFC, which is a heteropentameric AAA+ protein clamp loader for PCNA [13]. The ring-like PCNA homotrimer encircles DNA and slides spontaneously along the duplex as an essential subunit of the replisome [14]. RFC consists of the large subunit Rfc1 along with four smaller subunits: Rfc2, 3, 4 and 5. The smaller subunits are also present in alternative RFC-like complexes in which Rfc1 is replaced by Rad17, Ctf18 or Elg1 [15]. The Rad17-RFC complex has a well-characterized role in loading the Rad9-Hus1-Rad1 PCNA-like checkpoint clamp at DNA lesions and stalled replication forks, where it is essential for DNA damage and replication checkpoints enforced by Chk1 and Cds1/Chk2, respectively [16,17]. Ctf18 and Elg1 also play important but less well understood roles in maintaining genome integrity in response to replication-associated DNA damage [15,18].

As the *rfc3-1* mutation potentially impairs the functions of the canonical and alternative RFCs, we tested whether *htaAQ* has genetic interactions with *rad17∆*, *ctf18∆* or *elg1∆*. No obvious SSL interactions were detected (Fig 1B). To further test whether a defect in the canonical RFC creates a requirement for γH2A, we crossed *htaAQ* with the temperature sensitive *rfc1-44* mutation [15]. We detected a SSL interaction at 25°C that was enhanced at 32°C (Fig 1C). From these data we conclude that γH2A is crucial when the canonical RFC is impaired but not when the alternative RFC complexes are each individually ablated.

### Increased γH2A in *rfc3-1* cells

**Fig 2:**
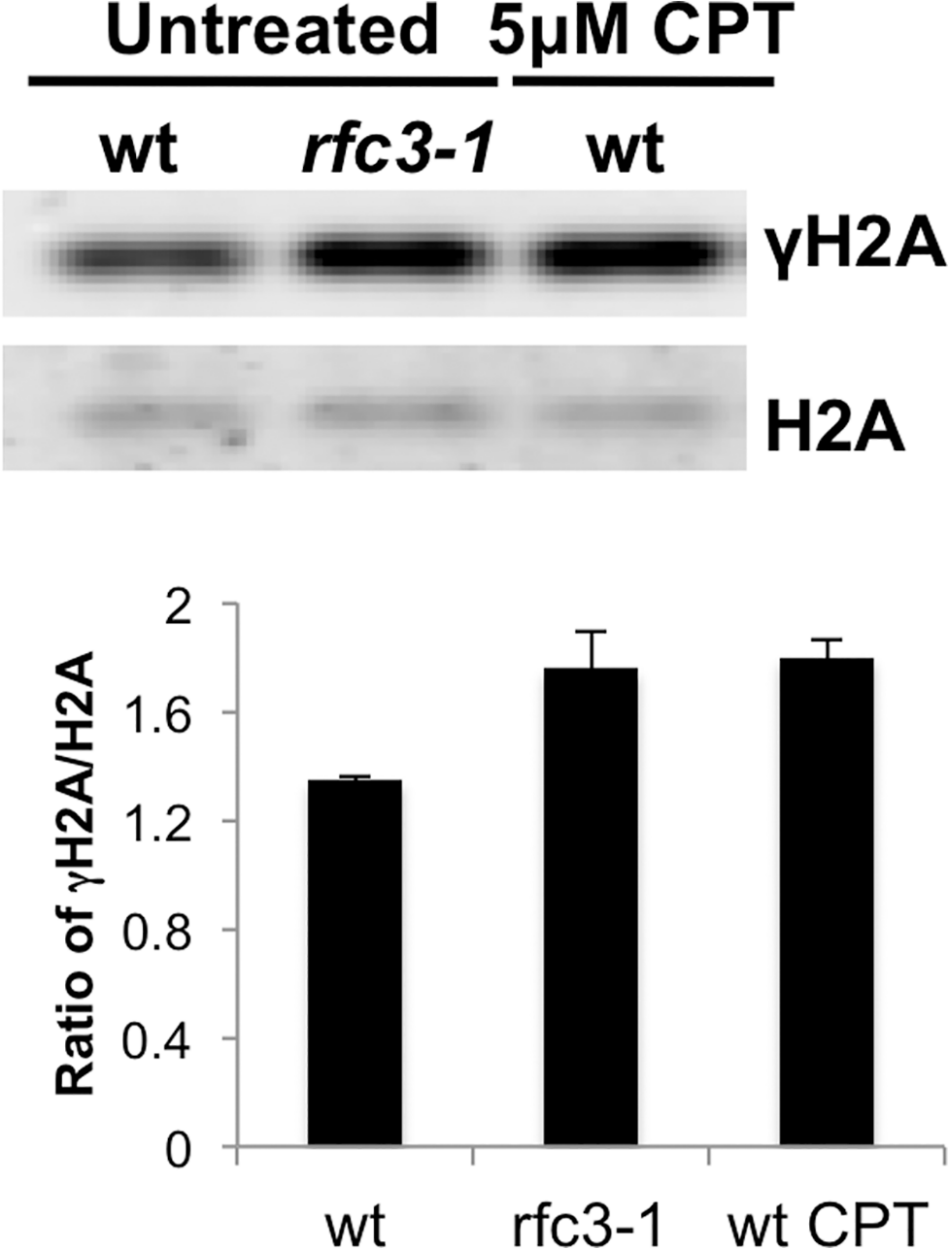
Increased γH2A in *rfc3-1* mutant. Histone enriched cell extracts from the indicated strains were immunoblotted with antisera that bind the C-terminal phospho-SQ epitope of γH2A or H2A itself. Note as shown below and reported previously γH2A in untreated wild type is predominantly from cells passing through S-phase [8]. Note also that *rfc3-1* cultures grown at 25°C were previously found to have a DNA content flow cytometry profile similar to wild type [12], indicating that increased γH2A in *rfc3-1* cultures most likely arises from increased γH2A-triggering lesions. The increased γH2A in *rfc3-1* cells cultured at 25°C is comparable to the level of γH2A in wild type cells treated with 5 μM CPT. Error bars indicate standard error of the mean of 3 independent experiments.

Our data suggested that replication defects in *rfc3-1* cells trigger a DNA damage response leading to formation of γH2A that is critical for maintaining viability. To test this idea we measured γH2A with anti-γH2A antisera [19] and found that it was increased in *rfc3-1* cells (Fig 2), matching the levels seen in wild type cells treated with the topoisomerase I poison camptothecin (CPT) that collapses replication forks [20].

### Brc1 binding to γH2A is crucial in *rfc3-1* cells

Crb2, Brc1 and Mdb1 bind γH2A in fission yeast [7,10,21,22]. Crb2 and Brc1 are most critical for surviving genotoxins [11,23,24], therefore we investigated the requirements for Crb2 and Brc1 in *rfc3-1* cells.

**Fig 3:**
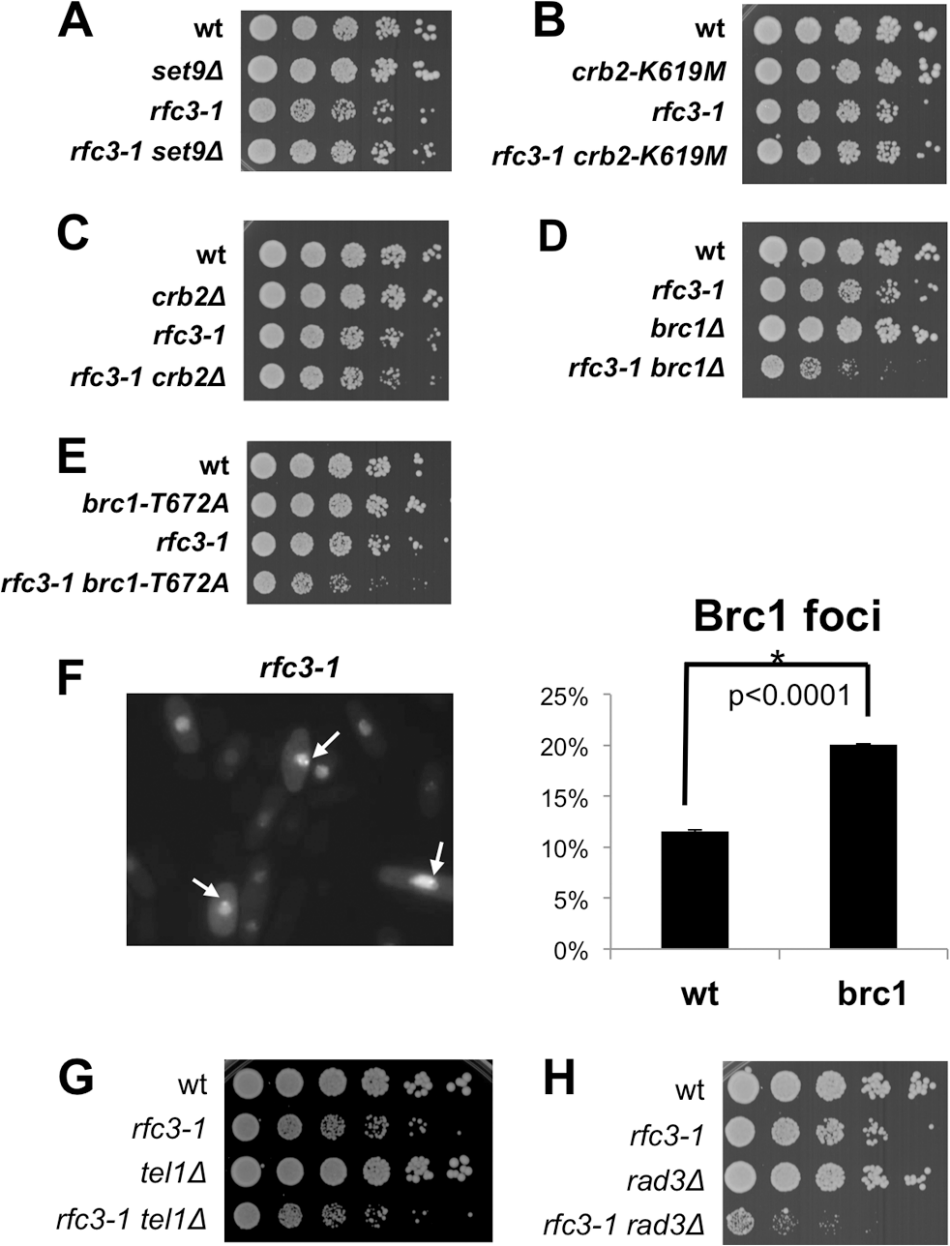
Brc1 binding to γH2A is critical in *rfc3-1* cells. All assays were performed at 25°C. **(A)** Elimination of histone lysine H4-K20 methyltransferase Set9, which creates a chromatin recruitment platform for Crb2, does not impair growth in *rfc3-1* cells. **(B)** The *crb2-K619M* mutation that ablates Crb2 binding to γH2A does not does not impair growth in *rfc3-1* cells. **(C)** Elimination of Crb2 weakly impairs growth in *rfc3-1* cells. **(D)** Elimination of Brc1 strongly impairs growth in *rfc3-1* cells. **(E)** The *brc1-T672A* mutation that ablates Brc1 binding to γH2A strongly impairs growth in *rfc3-1* cells. **(F)** Increased percentage of cells having GFP-Brc1 foci in *rfc3-1* cells incubated at 25°C. Arrows point to GFP-Brc1 foci. Error bars represent SEM from 3 experiments. **(G)** Eliminating Tel1 has little effect on the growth of *rfc3-1* cells. **(H)** Eliminating Rad3 strongly impairs growth of *rfc3-1* cells.

The tandem C-terminal BRCT domains of Crb2 that bind γH2A adjoin paired Tudor domains that bind dimethylated lysine-20 of histone H4 (H4-K20me2). Mutations that ablate these interactions are genetically epistatic and both interactions are required for large-scale localization of Crb2 at DSBs [25–27]. We found the elimination of the sole H4-K20 methyltransferase Set9 had no effect in *rfc3-1* cells (Fig 3A). Similarly, we found that *rfc3-1* cells were unaffected by the *crb2-K619M* mutation [26] that disrupts the γH2A-binding pocket (Fig 3B). As Crb2 retains partial function when γH2A and H4-K20me2 are simultaneously eliminated [26], we also tested the *crb2∆* mutation and found that it only weakly impaired growth in *rfc3-1* cells (Fig 3C). We conclude that Crb2 binding to γH2A and H4-K20me2 is not required in *rfc3-1* cells, while complete loss of Crb2 has a minor effect.

We next examined Brc1 and found that *brc1∆ rfc3-1* cells grew poorly compared to either single mutant (Fig 3D). We tested the *brc1-T672A* mutation that disrupts the γH2A binding pocket in Brc1 [10] and found a strong negative genetic interaction with *rfc3-1* (Fig 3E). These results established the importance of Brc1 binding to γH2A in *rfc3-1* cells.

### Increased Brc1 foci in *rfc3-1* cells

Our findings suggested that *rfc3-1* cells experience replication difficulties that trigger formation of γH2A and recruitment of Brc1 that is critical for survival. To further test this model we monitored formation of green fluorescent protein (GFP)-Brc1 foci, which increases in response to replication stress [10]. As predicted we detected a significant increase in GFP-Brc1 foci in *rfc3-1* cells incubated at 25°C (Fig 3F).

### Hus1-independent activity of Rad3/ATR is crucial in *rfc3-1* cells

Tel1/ATM and Rad3/ATR kinases create γH2A [7]. Eliminating Tel1 had no effect in *rfc3-1* cells (Fig 3G), which is consistent with Tel1 acting specifically at DSBs and telomeres as opposed replication forks [28,29]. In contrast, we detected a strong requirement for Rad3 in *rfc3-1* cells (Fig 3H), which supports evidence that Rad3 is critical for surviving replication stress [30].

**Fig 4:**
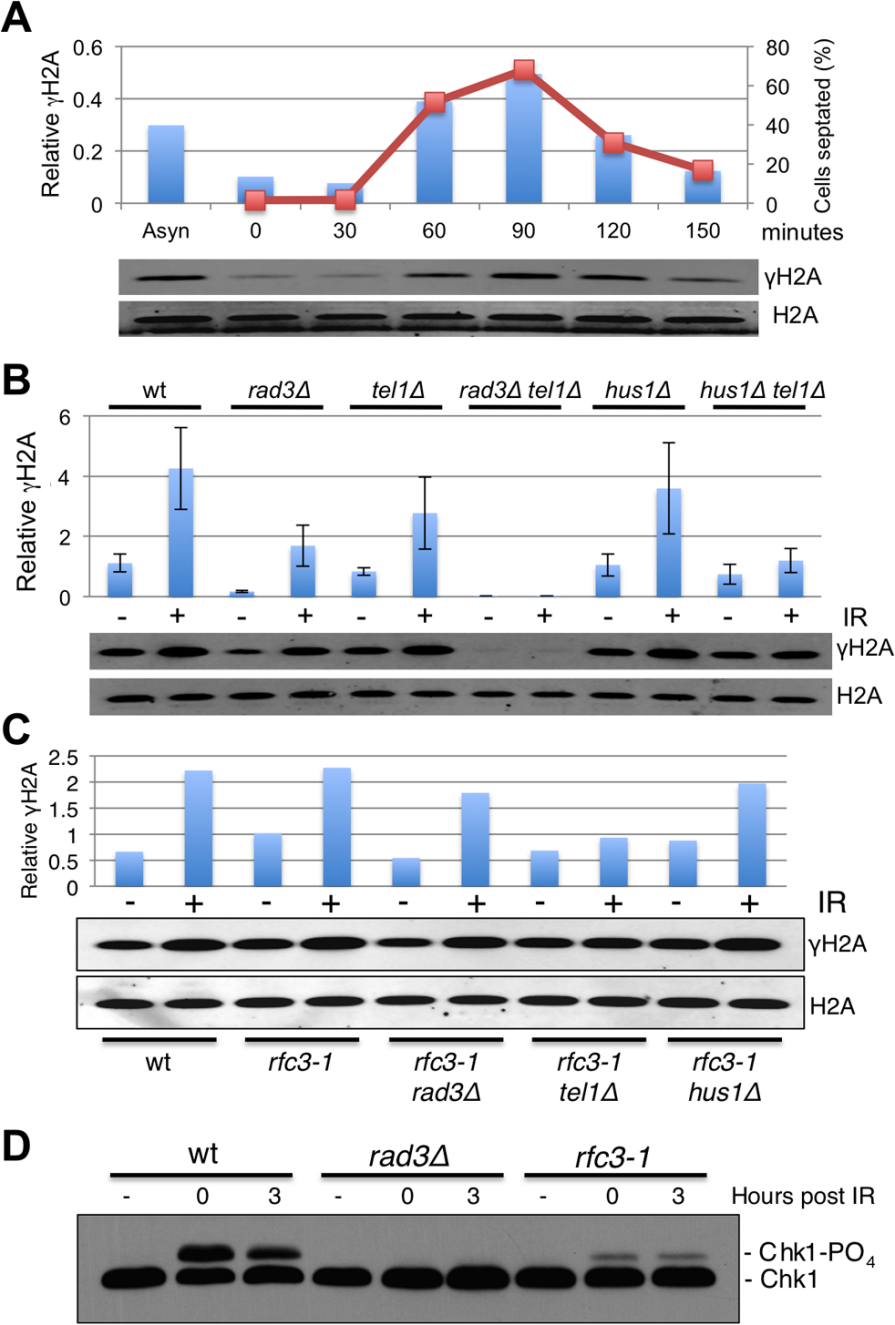
Hus1-independent phosphorylation of histone H2A by Rad3/ATR in *rfc3-1* cells. **(A)** In cells released from a *cdc25-22* late G2 phase cell cycle arrest, formation of γH2A (shown as bars) closely coincides with the increase in septation index (shown as line graph), which correlates with passage through S-phase. γH2A values were normalized to total H2A. **(B)** Immunoblot analysis with anti-γH2A antisera reveals that basal phosphorylation (-IR) of histone H2A by Rad3 does not depend on Hus1 (compare *hus1∆* to *hus1∆ tel1∆*). However, the IR-caused increase in γH2A in *hus1∆* cells is largely abolished in *hus1∆ tel1∆* cells, indicating that IR-induction of γH2A formation by Rad3 does require Hus1. Irradiated cells were harvested 30 minutes after 90 Gy of IR treatments. Values shown in graph were normalized to the total H2A signal. Error bars indicate standard error of the mean of 3 independent experiments. **(C)** The increase of γH2A in untreated *rfc3-1* cells does not depend on Hus1. **(D)** Rad3-dependent phosphorylation of Chk1 in response to IR is defective in *rfc3-1* cells.

Rad3 forms γH2A at stalled replication forks [8]. The dispensability of Rad17 in *rfc3-*1 cells suggested that Rad17-dependent loading of the Rad9-Hus1-Rad1 checkpoint clamp was not required for phosphorylation of H2A by Rad3 at stalled forks. This result was surprising because the Rad3 activator Cut5/Rad4 (TopBP1/Dpb11 ortholog) binds Rad9-Hus1-Rad1 [16,31]. We therefore investigated whether Rad9-Hus1-Rad1 regulates γH2A formation by Rad3 in S-phase. First, we used a synchronous culture to establish that γH2A in cycling cells occurs predominantly during S-phase (Fig 4A), confirming previous analyses performed by chromatin immunoprecipitation [8]. The large reduction of γH2A in untreated (-IR) *rad3∆* cells confirmed that Rad3 is principally responsible for forming γH2A during S-phase (Fig 4B). In contrast, the basal level of γH2A was maintained in *hus1∆* cells, showing that Rad3 activity towards histone H2A in S-phase does not require the checkpoint clamp (Fig 4B). Interestingly, eliminating Tel1 nearly abolished the IR-induced increase of γH2A in *hus1∆* cells, indicating that Rad3 activity towards histone H2A does require Hus1 at DSBs.

We also examined the genetic requirements for γH2A formation in *rfc3-1* cells grown at 25°C. In these assays the increase of γH2A in untreated *rfc3-1* required Rad3 but not Hus1 (Fig 4C), which is consistent with Rad3 but not Rad17 being required in *rfc3-1* cells (Figs 1B and 3H) Interestingly, IR induction of γH2A was largely abrogated in *rfc3-1 tel1∆* cells, indicating that phosphorylation of histone H2A by Rad3 at DSBs is decreased by *rfc3-1* at 25°C, presumably because of impaired loading of the Rad9-Hus1-Rad1 checkpoint clamp by Rad17-RFC. Indeed, Rad3-dependent phosphorylation of Chk1 was severely impaired in *rfc3-1* cells irradiated at 25°C (Fig 4D), mirroring previous studies performed at 28°C [12].

To summarize, the crucial phosphorylation of histone H2A by Rad3 during S-phase in *rfc3-*1 cells does not require the Rad9-Hus1-Rad1 checkpoint clamp, which explains why neither Rad17 nor Rfc3 are required for Rad3 activity towards histone H2A in *rfc3-*1 cells.

### Neither Cds1 nor Chk1 are required in *rfc3-1* cells

**Fig 5:**
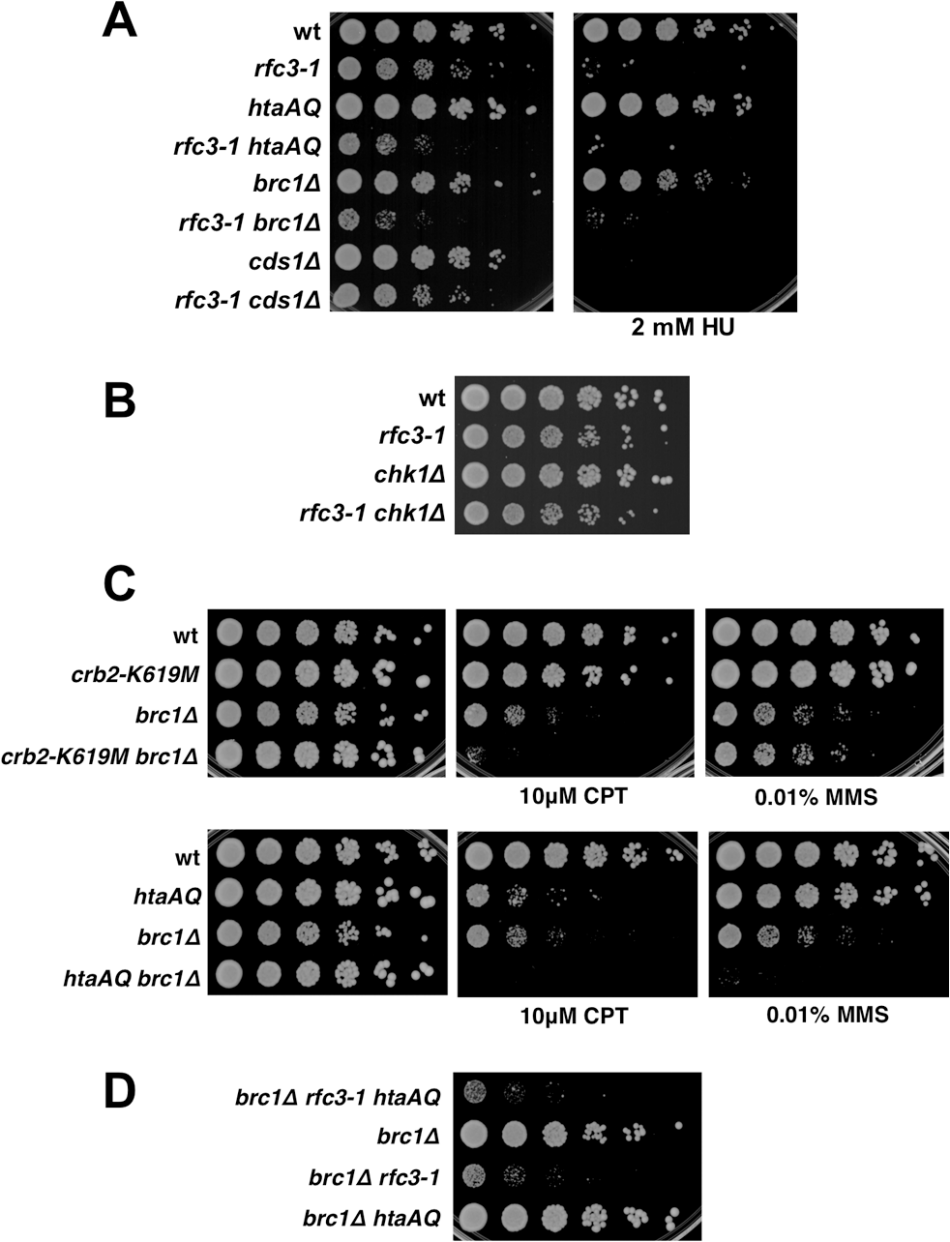
Chk1 and Cds1/Chk2 are not required for growth in *rfc3-1* cells, nor does Brc1 have an important checkpoint dampening function. **(A)** In contrast to eliminating γH2A or Brc1, deletion of Cds1 has little effect on growth in *rfc3-1* cells at 25°C. However, *cds1∆* are much more sensitive to HU. **(B)** Eliminating Chk1 has little effect on growth in *rfc3-1* cells at 25°C. **(C)** Neither *crb2-K619M* nor *htaAQ* suppress CPT or MMS sensitivity of *brc1∆* mutants, indicating that Brc1 does not have an important checkpoint dampening function. **(D)** Elimination of γH2A does not suppress the poor growth of *brc1∆ rfc3-1* cells.

Rad3 activates the checkpoint kinases Cds1/Chk2 and Chk1 by a mechanism that requires loading Rad9-Hus1-Rad1 checkpoint clamp onto DNA by Rad17-RFC [32]. Chk1 activation by Rad3 also requires Crb2. As Cds1 and Chk1 are amongst the most important and highly conserved Rad3 substrates it was surprising that neither Rad17 nor Crb2 are required in *rfc3-1* cells. We confirmed that neither Cds1 nor Chk1 are required in *rfc3-1* cells at 25°C (Fig 5A and 5B). The absence of a genetic interaction with *cds1∆* is especially notable because Cds1 is crucial for survival of hydroxyurea (HU) treatment, which stalls replication forks by inhibiting ribonucleotide reductase. Indeed, our spot dilution assays showed that *cds1∆* causes much greater HU sensitivity than *htaAQ* or *brc1∆* (Fig 5A). These data establish that very different DNA damage responses are required for survival of RFC defects and dNTP starvation, with the former requiring γH2A and the latter Cds1/Chk2 activation.

### Brc1 does not have an important checkpoint dampening function

The Brc1 structural homolog Rtt107 in *S*. *cerevisiae* competes with the Crb2 homolog Rad9 for binding γH2A to prevent hyper-activation of the checkpoint kinase Rad53 [33,34]. An equivalent activity might explain why Brc1 binding to γH2A is critical in *rfc3-1* cells. To test whether Brc1 has an important checkpoint dampening function we explored the effects of preventing Crb2 binding to γH2A in *brc1∆* cells. We found that the *crb2-K619M* mutation, which prevents Crb2 binding to γH2A [26], did not suppress the CPT or methyl methanesulfonate (MMS) sensitivity of *brc1∆* cells (Fig 5C). Indeed, *crb2-K619M* increased CPT sensitivity in the *brc1∆* background. Similarly, the *htaAQ* genotype increased both CPT and MMS sensitivity in *brc1∆* cells (Fig 5C). These data suggest that Brc1 is unlikely to have an important checkpoint dampening function in cells experiencing replication stress.

To investigate a potential anti-checkpoint activity of Brc1 in *rfc3-1* cells we constructed a *brc1∆ rfc3-1 htaAQ* strain. If Brc1 binding to γH2A is needed to dampen Crb2-dependent checkpoint signaling we would expect *htaAQ* to suppress the SSL interactions between *brc1∆* and *rfc3-1*. We observed no suppression; in fact, colony size appeared to be slightly smaller in *brc1∆ rfc3-1 htaAQ* cells compared to *brc1∆ rfc3-1* (Fig 5D).

Taken together these data indicate that Brc1 does not have an important checkpoint dampening function that could explain why *brc1∆* cells are sensitive to replication stress.

### Homologous recombination repair of collapsed replication forks is essential in *rfc3-1* cells

**Fig 6:**
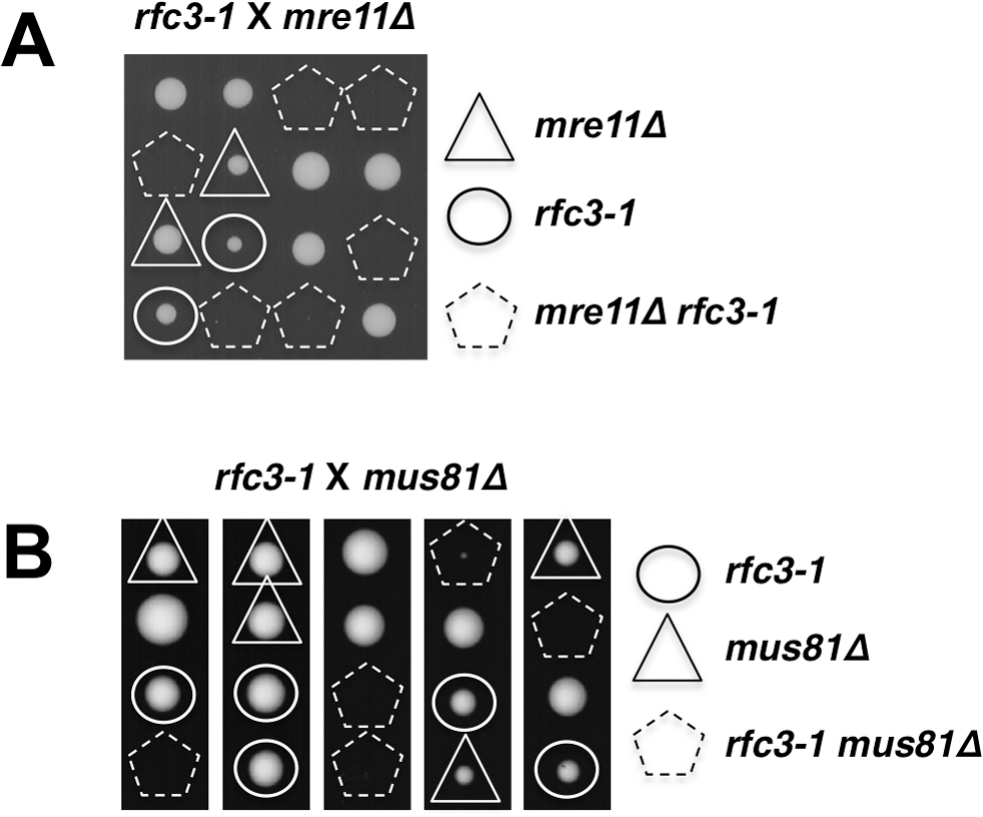
Mre11 and Mus81 are essential in *rfc3-1* cells. **(A)** Tetrad analysis reveals that *mre11∆ rfc3-1* cells are inviable at 25°C. **(B)** Tetrad analysis reveals that *mus81∆ rfc3-1* cells are inviable at 25°C.

Our data suggested that *rfc3-1* causes defects in DNA replication that may lead to the collapse of replication forks that are subsequently reestablished by homology directed repair (HDR) of the broken forks [35,36]. To investigate this possibility we first examined the Mre11-Rad50-Nbs1 (MRN) protein complex, which directly binds DSBs where it associates with Ctp1 to initiate 5’-to-3’ DNA end resection required for HDR [37,38]. Tetrad analysis revealed that *rfc3-1 mre11∆* cells are inviable at 25°C (Fig 6A).

Whereas MRN is required for HDR of all DSBs, Mus81-Eme1 endonuclease is specifically required to resolve Holliday Junctions created during HDR of one-ended DSBs formed by replication fork breakage [35,39]. We found that Mus81 is essential in *rfc3-1* cells germinated at 25°C, supporting the conclusion that the RFC defect in these cells leads to replication fork collapse (Fig 6B).

### Brc1 binding to γH2A suppresses catastrophic formation of ssDNA

**Fig 7:**
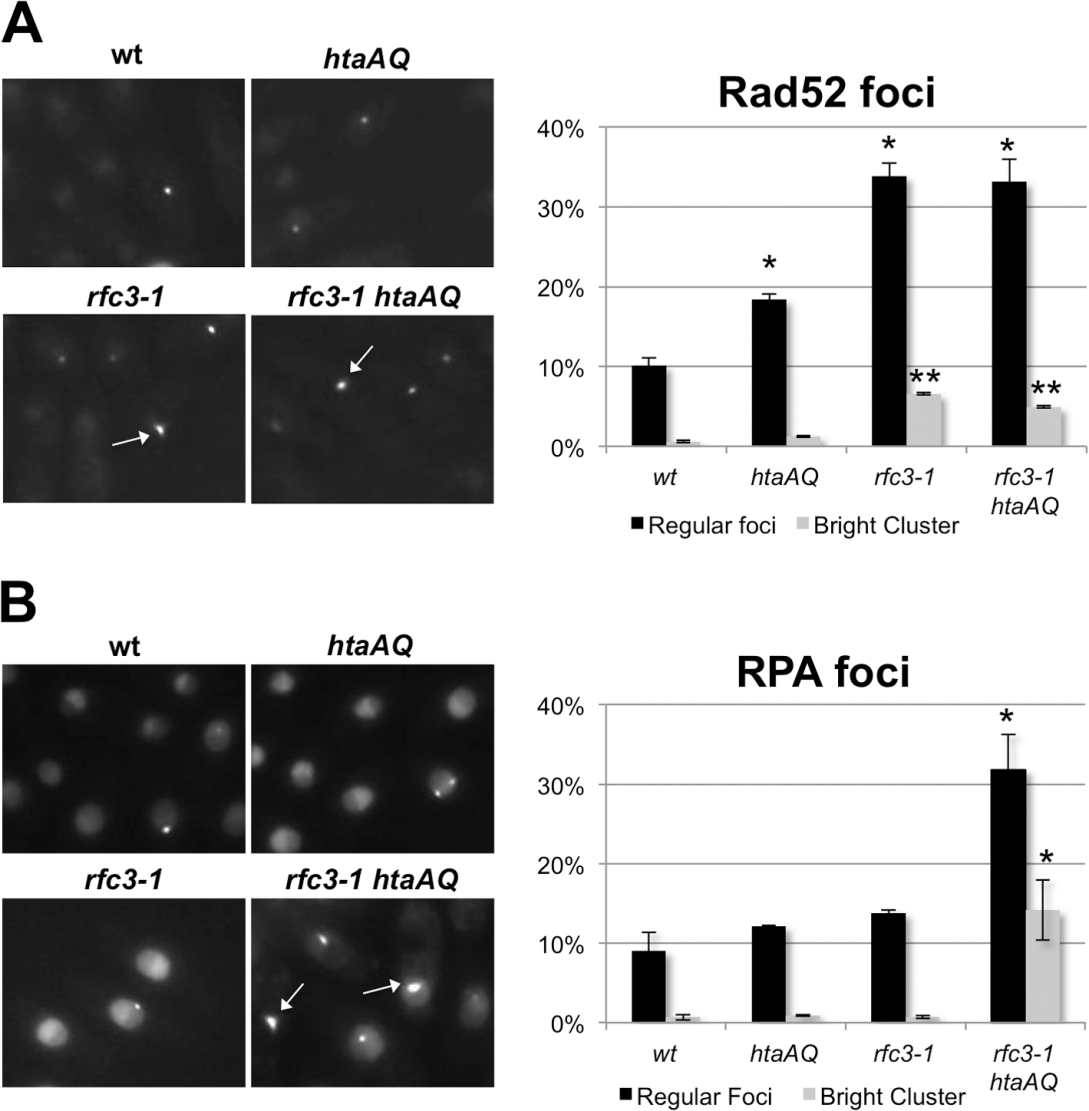
Loss of γH2A increases RPA foci in *rfc3-1* cells. **(A)** In comparison to wild type or *htaAQ* cells, *rfc3-*1 cells have many more nuclear Rad52 foci that often appear in clusters. However, eliminating γH2A had little effect on Rad52 foci in *rfc3-1* cells. Rad52-YFP foci were scored in live cells incubated at 25°C. Error bars represent SEM from 3 experiments. Single asterisk (*) indicates statistically significant difference (p-value <0.05) relative to wild type regular foci. Double asterisks (**) indicate statistically significant difference (p-value <0.001) relative to wild type bright cluster. P-values calculated using two-tailed unpaired T-test. **(B)** Eliminating γH2A in *rfc3-1* cells causes a large increase in nuclear RPA foci that often appear clustered. Ssb1-GFP was monitored in live cells incubated at 25°C. Arrows point to clusters of RPA foci. Error bars represent SEM from 3 experiments. Asterisk (*) indicates statistically significant difference (p-value <0.05) relative to corresponding measurements (regular foci or bright cluster) for wild type, *htaAQ* and *rfc3-1*. P-values calculated using two-tailed unpaired T-test.

Replication fork collapse is typically associated with nuclear foci formed by Rad52 HDR protein [40]. As predicted by our results, we detected a large increase in Rad52-yellow fluorescent protein (YFP) foci in *rfc3-1* cells grown at 25°C (Fig 7A). The *rfc3-1* strain further differed in having a significant percentage of cells with an unusually large and bright Rad52 focus that is likely clusters of Rad52 foci. However, eliminating γH2A did not substantially alter the Rad52 foci pattern of *rfc3-1* cells (Fig 7A).

We also monitored Ssb1 (aka Rad11), which is the largest subunit of Replication Protein A (RPA), the 3-subunit ssDNA-binding protein complex essential for DNA replication and most DNA repair mechanisms. RPA-green fluorescent protein (GFP) foci in *rfc3-1* cells appeared similar to wild type, indicating that in this situation Rad52 foci are better indicator of replication fork collapse. However, there was a large increase of RPA foci in *rfc3-1 htaAQ* cells (Fig 7B). Moreover, ~15% of the *rfc3-1 htaAQ* cells contained a very bright focus or cluster of RPA foci, which was rarely observed in wild type, *htaAQ* or *rfc3-1* cells. These results suggest Brc1 binding to γH2A suppresses catastrophic formation of ssDNA at replication forks in *rfc3-1* cells.

### γH2A is critical in a DNA polymerase epsilon mutant

**Fig 8:**
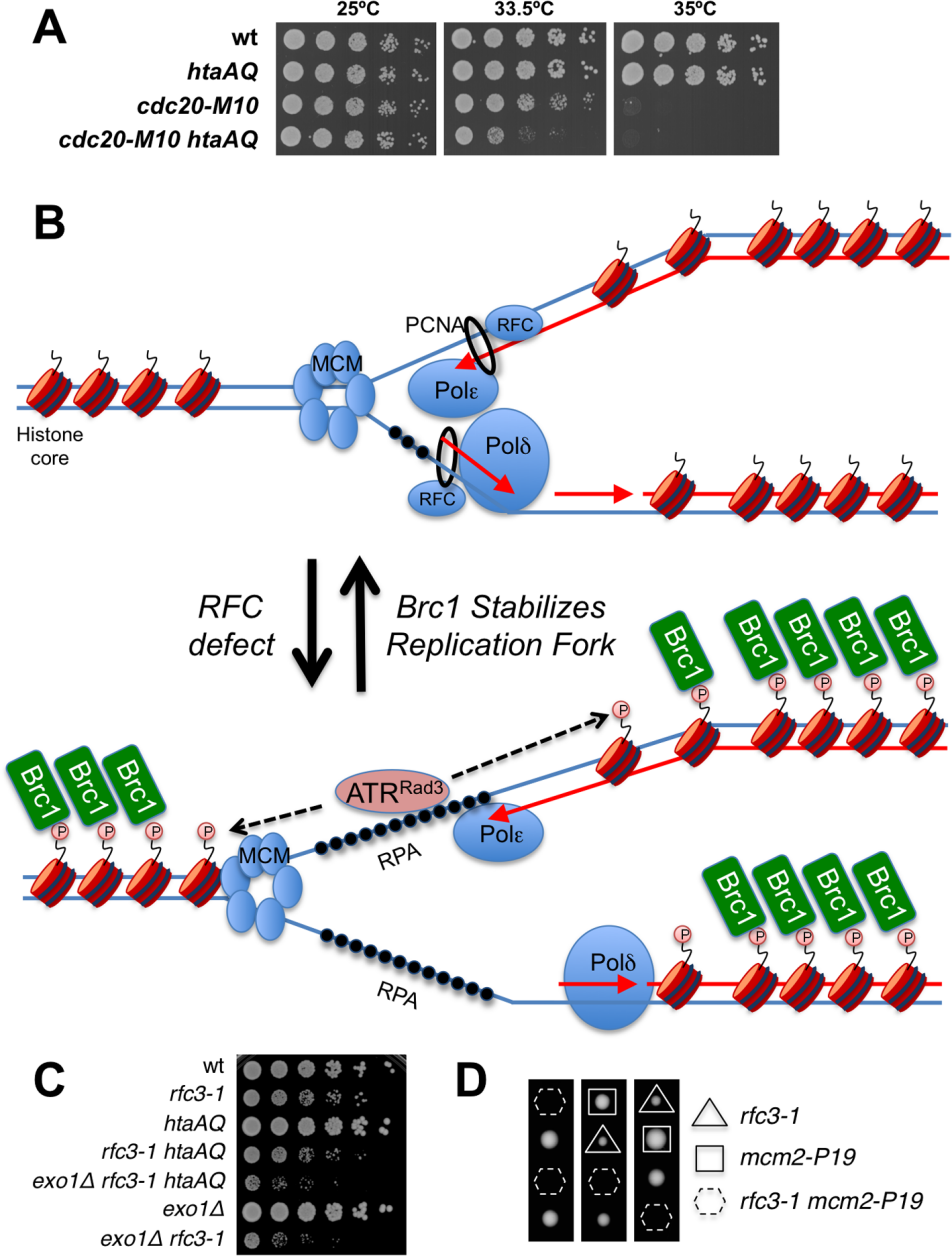
γH2A is critical in a DNA polymerase epsilon mutant. **(A)** Eliminating γH2A has a strong negative genetic interaction with the temperature sensitive *cdc20-M10* mutation of DNA polymerase epsilon in cells incubated at 33.5°C. **(B)** An RFC defect results in reduced loading of PCNA and poor coordination of MCM DNA helicase and DNA polymerases. Exposed ssDNA bound by RPA recruits ATR (ATR-ATRIP/Rad3-Rad26) that phosphorylates H2A without involving Rad17-RFC or the Rad9-Hus1-Rad1 checkpoint clamp. Brc1 binding to γH2A stabilizes the replisome at the replication fork. **(C)** Genetic interaction analyses showing that *exo1∆* mutation does not rescue poor growth of *rfc3-1 htaAQ* strain. **(D)** Tetrad analyses showing synthetic lethality between *rfc3-1* and *mcm2-P1*. Spores were germinated at 25°C.

RFC loads the PCNA clamp onto DNA, which facilitates the processivity of leading strand DNA replication through its interactions with DNA polymerase epsilon (Pol ε). We tested for genetic interactions between *htaAQ* and the *cdc20-M10* temperature sensitive mutation of Pol ε [41]. At the intermediate permissive temperature of 33.5°C we detected an acute requirement for γH2A in *cdc20-M10* cells (Fig 8A), mirroring the negative genetic interactions between *htaAQ* and *rfc3-1* or *rfc1-44* (Fig 1). These data indicate that a defect in tethering the leading strand DNA polymerase Pol ε at replication forks at least partially explains the requirement for γH2A in *rfc3-1* cells. Other deficiencies in *rfc3-1* cells, such as reduced tethering of additional DNA polymerases, might also be involved in creating the requirement for γH2A.

## Discussion

Phosphorylation of histone H2AX/H2A by ATM and ATR orthologs has long been known as a ubiquitous response to DSBs and was more recently uncovered as a response to replication stress, yet its physiological significance has remained unclear. The ATM/ATR-regulated checkpoint effector kinases Cds1/Chk2 and Chk1 are generally more important in clastogen and genotoxin sensitivity assays, as is Rad17 that is required for Cds1/Chk2 and Chk1 activation. In the same assays the γH2A-binding proteins Crb2 and Brc1 appear to have more crucial functions than γH2A itself [10,26]. The key discovery to emerge from these studies is that γH2A, and specifically Brc1 binding to γH2A, is critical when RFC is defective. By contrast, neither Cds1 nor Chk1 are required in this situation. Similarly, ablating the Rad17-dependent Rad9-Hus1-Rad1 clamp loader causes acute genotoxin sensitivity but has little effect in *rfc3-1* cells. This complete reversal of DDR mutant sensitivities when comparing *rfc3-1* to exogenous DNA damaging agents and replication inhibitors is striking.

The picture that emerges from these studies is that most genotoxins fail to replicate the effects of impairing RFC, which presumably reduces PCNA loading and DNA polymerase tethering at the replication fork. This idea is supported by the requirement for γH2A when Pol ε is partially impaired, although the genetic interactions involving *rfc3-1* and *cdc20-M10* are not precisely identical [41]. If DNA polymerase activity is a rate-limiting step for DNA replication under some physiological conditions our studies suggest that Brc1 binding to γH2A is critical for protecting genome integrity in these situations. This model is consistent with increased Rad52 foci in *htaAQ* and *brc1∆* cells [10,11].

Our findings call to mind a study in which reduced levels of DNA polymerase alpha were found to trigger chromosome translocations in budding yeast [42]. These translocations involved HDR events between long terminal repeats (LTRs) of Ty retrotransposons elements, which were proposed to be chromosome fragile sites. Another study found that LTRs are specifically enriched for γH2A during S-phase [9], as was also observed in fission yeast [8]. Our studies suggest that besides marking DSBs arising at chromosome fragile sites, γH2A also serves to stabilize replisomes at these sites and thereby prevent chromosome breakage, perhaps by binding the Brc1 structural homolog known as Rtt107 in budding yeast [43,44].

The unusual RPA foci in *rfc3-1 htaAQ* cells suggest to us either catastrophic DNA unwinding or massive resection of DSBs. Both events may occur but we favor the idea that DNA unwinding uncoupled from DNA synthesis most likely explains the critical requirement for γH2A in RFC and Pol ε defective cells (Fig 8B). We favor this model because elimination of Exo1 exonuclease, which is primarily responsible for long-range resection in fission yeast [37], does not suppress the poor growth of *rfc3-1 htaAQ* cells (Fig 8C). To the contrary, the *exo1∆* mutation further impairs the growth of *rfc3-1 htaAQ* cells. This effect can be explained by a requirement for Exo1 in *rfc3-1* cells regardless of whether these cells are able to form γH2A (Fig 8C). This genetic interaction might indicate that Exo1 contributes to HDR of broken replication forks in *rfc3-1* cells, although we note that *exo1∆* cells are largely insensitive to IR, CPT and MMS, all of which cause DNA damage that is repaired via HDR [45,46].

The massive accumulation of RPA foci in *rfc3-1 htaAQ* cells calls to mind a recent study with mammalian cells in which ATR was found to prevent global exhaustion of RPA to prevent replication catastrophe [47]. In this study ATR was proposed to prevent RPA exhaustion by restraining origin firing. Our studies suggest that stabilization of stalled replication forks may also play a role in this process, perhaps involving formation of γH2AX. The requirement for Mus81 in *rfc3-*1 cells suggests that Brc1 binding to γH2A does not completely prevent replication fork collapse. Brc1 binding to γH2A likely facilitates repair of broken replication forks, thereby compounding the requirement for γH2A in *rfc3-1* cells. This proposal comports with the evidence that *htaAQ* mutants are sensitive to camptothecin [7,19]. We also note that post-translational modifications of PCNA promote post-replication repair (PRR) of DNA lesions [48,49]. Brc1 was proposed to function in conjunction with PRR proteins, including components of the HDR machinery, as well as with multiple structure-specific nucleases [11,50,51]. Thus a defect in PRR might also contribute to the requirement for γH2A in *rfc3-1* cells, although it remains to be established whether Brc1 binding to γH2A promotes PRR.

If defective loading of PCNA leads to DNA polymerase uncoupling from MCM DNA helicase in *rfc3-1 htaAQ* cells it might be possible to suppress this defect by using a temperature sensitive mutation to partially impair MCM activity. We attempted this experiment with the *mcm2-P1* (aka *cdc19-P1*) allele. Although we found that eliminating γH2A had no effect on the growth of *mcm2-P1* cells at 25°C, combining *htaAQ mcm2-P1* with *rfc3-1* resulted in synthetic lethality. In an independent experiment we confirmed that *mcm2-P1 rfc3-1* cells were inviable at 25°C (Fig 8D).

ATM and ATR use γH2AX to effect chromatin-specific responses to DNA damage. Multi-kilobase γH2AX domains have been detected in yeast and megabase domains in mammals [3]. Why are these responses so highly conserved? In fission yeast, coating chromatin with Crb2 likely serves to rapidly amplify and reliably maintain Chk1 activity during DSB repair [26]. These properties may be most critical when cells suffer a single DSB, which is the most common situation for endogenous sources of DSBs. The purpose of γH2A at stalled or damaged replication forks is probably quite different. From the insights provided by the current study we propose that mounting large-scale changes in chromatin by decorating it with Brc1 is well suited for coordinating the activities of the replicative DNA helicase with leading and lagging stand DNA polymerases (Fig 8B).

It remains unclear whether Brc1 activities are conserved with its structural homologs Rtt107 in budding yeast or PTIP in mammals [52,53]. Rtt107 binding to γH2A was most recently shown to be important for assembling Slx4 signaling protein complexes behind damaged replication forks [54]. However, this signaling activity does appear to be conserved in fission yeast, in which Slx4 function appears to solely involve forming an active structure-specific endonuclease with Slx1 [55,56]. Furthermore, the checkpoint dampening activity of Rtt107 [33,34] does not appear to be conserved in fission yeast or at least is not detectably important in our genetic assays (Fig 5). The function of PTIP, which shares the 6 BRCT domains arrangement with Brc1 and Rtt107, the C-terminal pair of which bind γH2AX [57], is also a matter of substantial interest. Functional relationships of Brc1 or Rtt107 to PTIP are currently unobvious. Recent studies suggest that PTIP functions with 53BP1 in inhibiting HDR [58–60]. Perhaps Brc1, Rtt107 and PTIP all modulate DNA end resection with varying effects, although we note the genetic interactions involving Exo1 do not support this idea for Brc1 (Fig 8C). Functional similarities may emerge with new functional insights into this class of genome protection proteins.

## Materials and Methods

### General methods

Standard genetic procedures and media for *S*. *pombe* were used as described [61]. Strains expressing GFP-Brc1 were constructed by inserting *Mlu*I digested pREP41-GFP-Brc1 [10] into the *ars1* locus. For spot dilution assays log phase cultures were suspended at 0.4 OD600 and serially diluted five-fold onto YES (yeast extract, glucose and supplements) agar plates. Cell survival was determined after 5 days at 25°C, 3–4 days at 30°C and 2 days at temperatures over 30°C. Strains used in this study are listed in S1 Table.

### Immunoblot analysis

The γH2A immunoblots were performed using acid protein extraction to obtain histone-enriched extracts [19] in Fig 2 or total cell extracts in Fig 4. Proteins were resolved by SDSPAGE on 4–20% tris-glycine gels (Life Technologies). Blocking and blotting were performed with Odyssey Blocking Buffer (Li-Cor) per manufacturer instructions and incubated with a rabbit polyclonal phospho-specific anti-γH2A antibody (courtesy of C. Redon). Total H2A was detected using polyclonal anti-H2A antibody 07–146 Millipore for Fig 2 or Active Motif 39235 for Fig 4. Blots were incubated with goat anti-rabbit antibody conjugated to an infrared dye (Li-Cor 827–11081) and scanned and quantified with Odyssey Infrared Imaging System (Li-Cor) with an intensity of 4.5, subtracting median (top/bottom) background.

### Microscopy

Cells were photographed using a Nikon Eclipse E800 microscope equipped with a Photometrics Quantix CCD camera and IPlab Spectrum software. Rad52-YFP and Ssb1-GFP were expressed from endogenous loci. Ssb1 (aka Rad11) is the largest subunit of RPA. Rad52-YFP and Ssb1-GFP experiments used cells grown in YES at 25°C. GFP-Brc1 was expressed from the *nmt1* promoter using EMM2 (Edinburgh Minimal Media) without thiamine. At least 300 nuclei were scored in three independent experiments. All microscopy was conducted with live (unfixed) cells.

## Supporting Information

S1 Table Strains used in this study. All strains are *leu1-32 ura4-D18* unless otherwise noted. Strains listed as *his3* may contain *his3-D1*. *his7* may contain *his7-336*.
(DOCX)
