# Supplementary material for: Critical Function of γH2A in S-Phase
Source: PLoS Genet. 2015 Sep 14;11(9):e1005517. doi: 10.1371/journal.pgen.1005517 (PMC4569340; doi:10.1371/journal.pgen.1005517)
Supplement: S1 Table — All strains are leu1-32 ura4-D18 unless otherwise noted. Strains listed as his3 may contain his3-D1. his7 may contain his7-336. (DOCX) [file pgen.1005517.s001.docx]

**Supplementary Table 1. Strains used in this study.** All strains are *leu1-32 ura4-D18* unless otherwise noted. Strains listed as *his3* may contain *his3-D1.*  *his7* may contain *his7-336.*

| **Strain number** | **Genotype** | **Source** |
| --- | --- | --- |
| PR109 | *h^-^* | Lab strain |
| EM5230 | *ura4^+^ h^+^* | Lab strain |
| MSY1 | *rfc3-1 his7-336 h^+^* | [1] |
| SR139 | *hta1-S129A:ura4^+^ hta2-S128A:kanMX6 h^+^* | [2] |
| SR155 | *hta1-S129A:ura4^+^ hta2-S128A:kanMX6 h^-^* | [2] |
| EM5231 | *rfc3-1 hta1-S129A:ura4^+^ hta2-S128A:kanMX6 his7-336 h^-^* | This study |
| EM5232 | *rfc3-1 hta1-S129A:ura4^+^ hta2-S128A:kanMX6 his7 h^+^* | This study |
| JW4724 | *brc1::hph h^+^* | [3] |
| EM5233 | *rfc3-1 brc1::hph his7-336 h^+^* | This study |
| EM5234 | *crb2::ura4^+^ his7* | This study |
| EM5235 | *rfc3-1 crb2::ura4^+^ his7* | This study |
| EM5236 | *set9::kanMX6 his7* | This study |
| EM5237 | *rfc3-1 set9::kanMX6* | This study |
| EM5238 | *brc1-T672-2GFP:hph his3 his7* | This study |
| EM5239 | *rfc3-1 brc1-T672-2GFP:hph his3 his7 h^-^* | This study |
| EM5240 | *brc1-2GFP::hph his3 his7* | This study |
| EM5241 | *leu1-32::2CFP-crb2-K619M-leu1^+^ crb2::nat his3* | This study |
| EM5242 | *rfc3-1 leu1-32::2CFP-crb2-K619M-leu1^+^ crb2::nat his3* | This study |
| EM5243 | *rfc3-1 brc1-2GFP::hph ura4^+^* | This study |
| EM5244 | *rfc3-1 hta1-S129A:ura4^+^ hta2-S128A:his3^+^ brc1-T672-2GFP:hph his3 his7* | This study |
| JW4744 | *hta1-S129A:ura4^+^ hta2-S128A:his3^+^ brc1-T672-2GFP:hph his3 his7 h-* | Lab strain |
| EM5245 | *ars1::Pnmt1-GFP-brc1^+^-LEU2 h^+^* | This study |
| EM5246 | *rfc3-1 ars1::Pnmt1-GFP-brc1^+^-LEU2 his7-366 h^+^* | This study |
| TMN3333 | *rad52-YFP:kanMX6 his3-D1* | [4] |
| EM5247 | *hta1-S129A:ura4^+^ hta2-S128A:kanMX6 rad52-YFP:kanMX6* | This study |
| EM5248 | *rfc3-1 rad52-YFP:kanMX6 his3 his7* | This study |
| EM5249 | *rfc3-1 hta1-S129A:ura4^+^ hta2-S128A:kanMX6 rad52-YFP:kanMX6 his3 his7 h^-^* | This study |
| EM4889 | *rad11-GFP(S65T):hph h^+^* | [5] |
| EM5250 | *hta1-S129A:ura4^+^ hta2-S128A:kanMX6 rad11- GFP(S65T):hph h^-^* | This study |
| EM5251 | *rfc3-1 hta1-S129A:ura4^+^ hta2-S128A:kanMX6 rad11- GFP(S65T):hph his3 his7* | This study |
| TMN3518 | *rad3::kanMX4 ade6-M216 his3-D1 h^-^* | Lab strain |
| EM5252 | *rfc3-1 rad3::kanMX4 ura4-D18 ade6-M216? his3 his7* | This study |
| EM5253 | *rfc3-1 tel1::kanMX4 ade6-M210? his3 his7* | This study |
| EM5254 | *rfc1-44 h^+^* | [6] |
| EM5255 | *rfc1-44 leu1-32 h^-^* | This study |
| EM5256 | *rfc1-44 hta1-S129A:ura4^+^ hta2-S128A:kanMX6 ura4^?^ h^+^* | This study |
| YY4318 | *cdc20-M10 ura4^+^ h^-^* | Lab strain |
| EM5257 | *cdc20-M10 hta1-S129A:ura4^+^ hta2-S128A:kanMX6 h^-^* | This study |
| TMN2799 | *mre11::kanMX4 ade6-M210 his3-D1 h^-^* | [7] |
| PR4053 | *ctf18::his3^+^ his3-D1 h^?^* | Lab strain |
| EM5258 | *ctf18::his3^+^ hta1-S129A:ura4^+^ hta2-S128A:kanMX6 ade6-210 his3-D1* | This study |
| EM5259 | *elg1::kan hta1AQ::ura4 hta2-S128A:kanMX6* | This study |
| TMN2936 | *rad17::ura4 ade6-M216 his3-D1 h^+^* | Lab strain |
| EM5260 | *rad17::ura4^+^ hta1-S129A:ura4^+^ hta2-S128A:kanMX6 his3-D1 h^-^* | This study |
| OM810 | *ura4^+^ h^-^* | Lab strain |
| TMN3291 | *hta1-S129A:ura4^+^ hta2-S128A:his3^+^ his3-D1 h^+^* | [4] |
| EM5261 | *elg1::kan ura4^+^ h^-^* | This study |
| OL5262 | *rfc3-1 h^-^* | This study |
| OL5263 | *tel1::hphMX6 h^-^* | This study |
| OL5264 | *tel1::hphMX6 rfc3-1 h^-^* | This study |
| TMN2665 | *ade6-M210 his3-D1 h^-^* | [7] |
| TMN2937 | *rad3::ura4^+^ ade6-M210 his3-D1 h^-^* | [7] |
| TMN2967 | *tel1::kanMX4 ade6-M210 his3-D1 h^-^* | [7] |
| TMN2978 | *tel1::kanMX4 rad3::ura4 ade6-M210 his3-D1 h^?^* | [7] |
| TMN2933 | *hus1::LEU2 ade6-M216 his3-D1 h^-^* | [7] |
| TMN2974 | *tel1::kanMX4 hus1::LEU2 ade6-M210 his3-D1 h^-^* | [7] |
| KS1483 | *cdc25-22 h^+^* | Lab strain |
| OL5352 | *rfc3-1 rad3::kanMX4 h-* | This study |
| OL5353 | *rfc3-1 hus1::LEU2 his3 h-* | This study |
| LLD3427 | *chk1-9myc-2HA6his:ura4+ h-* | [8] |
| OL4849 | *rad3::kanMX4 chk1-9myc-2HA6his:ura4+ h-* | [9] |
| OL5354 | *rfc3-1 chk1-9myc-2HA6his:ura4+ h-* | This study |
| TMN4887 | *hta1-S129A:ura4^+^ hta2-S128A:kanMX4 his3-D1 h^+^* | [4] |
| EN3169 | *brc1::kanMX6 h^+^* | Lab strain |
| OL4857 | *cds1::hphMX6 h^-^* | [9] |
| EM5265 | *rfc3-1 cds1::hphMX6 h^?^* | This study |
| OL4033 | *chk1::hphMX6 h^-^* | This study |
| OL5266 | *rfc3-1 chk1::hphMX6 h^-^* | This study |
| EN3190 | *mus81::kanMX h^+^* | Lab strain |
| EM5351 | *rfc3-1 rad11- GFP(S65T):hph his3 his7 h^-^* | This study |
| LLD4902 | *leu1-32::2xYFP-crb2-K619M-leu1+ crb2::ura4+ his3-D1 h-* | [10] |
| JW4718 | *brc1::hphMX6 h-* | This study |
| SAS4924 | *leu1-32::2xYFP-crb2-K619M-leu1+ crb2::ura4+ brc1::hphMX6* | [10] |
| JW4715 | *hta1-S129A:ura4^+^ hta2-S128A:his3 his3-D1 brc1::kanMX6 h-* | This study |
| EM5355 | *rfc3-1 hta1-S129A:ura4^+^ hta2-S128A:his3 his3-D1 brc1::hphMX6 his3-D1* | This study |
| OL5356 | *rfc3-1 brc1::kanMX6t h-* | This study |
| OL5357 | *rfc3-1 exo1::natMX6 hta1-S129A:ura4 hta2-S128A:kanMX4 rad11-GFP:hphMX6 h-* | This study |
| OL4175 | *exo1::ura4+ h-* | [11] |
| OL5358 | *rfc3-1 exo1::natMX6 rad11-GFP:hphMX6* | This study |
| PR1851 | *mcm2-P19* | Lab strain |

**References**

1. Shimada, M., et al., *Replication factor C3 of Schizosaccharomyces pombe, a small subunit of replication factor C complex, plays a role in both replication and damage checkpoints.* Mol Biol Cell, 1999. **10**(12): p. 3991-4003.

2. Rozenzhak, S., et al., *Rad3 decorates critical chromosomal domains with gammaH2A to protect genome integrity during S-Phase in fission yeast.* PLoS Genet, 2010. **6**(7): p. e1001032.

3. Williams, J.S., et al., *gammaH2A binds Brc1 to maintain genome integrity during S-phase.* EMBO J, 2010. **29**(6): p. 1136-48.

4. Nakamura, T.M., et al., *Histone H2A phosphorylation controls Crb2 recruitment at DNA breaks, maintains checkpoint arrest, and influences DNA repair in fission yeast.* Mol Cell Biol, 2004. **24**(14): p. 6215-30.

5. Cavero, S., O. Limbo, and P. Russell, *Critical functions of Rpa3/Ssb3 in S-phase DNA damage responses in fission yeast.* PLoS Genet, 2010. **6**(9): p. e1001138.

6. Kim, J., et al., *Contrasting effects of Elg1-RFC and Ctf18-RFC inactivation in the absence of fully functional RFC in fission yeast.* Nucleic Acids Res, 2005. **33**(13): p. 4078-89.

7. Nakamura, T.M., B.A. Moser, and P. Russell, *Telomere binding of checkpoint sensor and DNA repair proteins contributes to maintenance of functional fission yeast telomeres.* Genetics, 2002. **161**(4): p. 1437-52.

8. Du, L.L., B.A. Moser, and P. Russell, *Homo-oligomerization is the essential function of the tandem BRCT domains in the checkpoint protein Crb2.* J Biol Chem, 2004. **279**(37): p. 38409-14.

9. Limbo, O., et al., *Mre11 nuclease activity and Ctp1 regulate Chk1 activation by Rad3ATR and Tel1ATM checkpoint kinases at double-strand breaks.* Mol Cell Biol, 2011. **31**(3): p. 573-83.

10. Sofueva, S., et al., *BRCT domain interactions with phospho-histone H2A target Crb2 to chromatin at double-strand breaks and maintain the DNA damage checkpoint.* Mol Cell Biol, 2010. **30**(19): p. 4732-43.

11. Williams, R.S., et al., *Mre11 dimers coordinate DNA end bridging and nuclease processing in double-strand-break repair.* Cell, 2008. **135**(1): p. 97-109.
